# Supplementary material for: Patterns of Intron Gain and Loss in Fungi
Source: PLoS Biol. 2004 Nov 30;2(12):e422. doi: 10.1371/journal.pbio.0020422 (PMC532390; doi:10.1371/journal.pbio.0020422)
Supplement: Table S1 — Also available at http://genes.mit.edu/NielsenEtAl/. (4.3 MB ZIP). [file pbio.0020422.st001.zip › NielsenEtAl/html/1298.html]

AN5725.1.NCU06779.1.MG03883.1.FG10858.1


```
 CLUSTAL W (1.82) Multiple Sequence Alignments - Introns Inserted


Sequence 1: NCU06779.1	556 aa
Sequence 2: MG03883.1	619 aa
Sequence 3: FG10858.1	530 aa
Sequence 4: AN5725.1	585 aa
Alignment Length: 714 aa
Number Identitical Residues: 214 aa
Alignment Score (without introns) 11622


MG03883.1 	MERPCLFGRDCAAAGKNIWPALRVLLPVGLVRSMRPLAYSYCTNRAL0GIQFSCCSLPPR
NCU06779.1	------------------------------------MANGLFTYVAI~------------
FG10858.1 	-----------------------------------------------~------------
AN5725.1  	---------------------------------MLFLLKFLIS----~------------
          	                                          :                 

MG03883.1 	AGSPSTLLPYSRPPSLWTIAKPARLSLPPSQPAMDGFGDSDKCASGSTSSLSAGSSCQDS
NCU06779.1	--------------SLFTIG----------------------------------------
FG10858.1 	------------MPSLTALV----------------------------------------
AN5725.1  	------------VIALLAAC----------------------------------------
          	              :* :                                          

MG03883.1 	QGSYSLYATFTTIAAMPLLYFFIVPWFWRAIGSALGWYLKRKTEGRRAQLLEIMNEDERR
NCU06779.1	----------------PLLALFIP-FVWRLVGSSLGWYLRRKTDGRRCHILEVVEADERK
FG10858.1 	----------------PLAILIAIPLLGKFLRTFLGWSLQKRTEGRRAHLLALMTEEDR-
AN5725.1  	----------------VLLPQFPLGLLRIVLR-GVGWVIRKRTQARREVILSRVRADEDE
          	                 *   :    .   :   :** ::::*:.**  :*  :  ::  

MG03883.1 	FAESGQ----ERKKSVTDEEWESIETPAGSADNGGKA-------AQEWDGIVGFFHPFC2
NCU06779.1	YRDSKS----AKGRKAEKEDRDTFNNTEATGTSDGKVY------DKDWDGIVGFFHPFC2
FG10858.1 	AAHSRD----PRGSAETKLVFDVDDNLQNTLSS-----------QKDWAGIVGFFHPFC2
AN5725.1  	LLSKQSKVACTSTTSADDEDWEKVDSSSGDSSSGSASQNFQQSGTDEWTGIIGFFHPFC2
          	   . .. :.       .   :  :.      ...   . ..:. .:* **:******* 

MG03883.1 	NAGGGGERVLWAAIRATQQKWPKAKCIVYTGDHEVNKEAILSRVE~V-------------
NCU06779.1	NAGGGGERVLWAAIRATQKRWPKAKCVVYTGDHDVSKEAILSRVE0QRFNIHLHPPTVNF
FG10858.1 	NAGGGGERVLWAAIRATQDRWPKAKCVVYTGDHDVTKDAILSRVK0TRFNIELHAPTMTF
AN5725.1  	NAGGGGERVLWEAVRATQRRWPKAVCAIYTGDLNVTKAAMLERVQ0TRFNIRLHAPTVEL
          	*********** *:**** :**** * :**** :*.* *:*.**:    .    ..:   

MG03883.1 	-----------------------------S1WDAFSLLTPDIFVDTMGYAFALGLCRWLF
NCU06779.1	LYLSTRRWVLASTWPYFTLAGQSFGSLIMA~WDAFSLLVPDIFVDTMGYAFALGFSRFLF
FG10858.1 	LYLSKRDWVLPSTWPHFTLLGQSLGSVVLA~WDAFSLLVPDVFIDTMGYAFALGLCKFLF
AN5725.1  	LYLTTRKYVQSSMYPYMTLLGQSLGSLIVG~YDAFTLLVPDVFVDTMGYAFAVAFCKLLF
          	   :.     .:  .  :  ..: .:   . :***:**.**:*:********:.:.: **

MG03883.1 	HTMPTGAYVHYPTISTDMLDSLNPHSAVGSQGVNAGQGVGARGRAKKLYWELFAKIYCRM
NCU06779.1	RDVPTAAYVHYPTISTDMLESLDPASAVGSQGVNAGKGTGAKGRAKKIYWQLFARLYSLM
FG10858.1 	PKIPTGAYVHYPTISTDMLESLDVKEDAGTQGAHVGKGAGAQGFAKKNYWKLFAVIYSWV
AN5725.1  	PSVPTGAYVHYPTISTDMLQSLD--DKTGLKGVNAGAGTGLKGTLKRKYWLAFAHLYGWI
          	  :**.*************:**:  . .* :*.:.* *.* :*  *: **  ** :*  :

MG03883.1 	GATIDVVMTNSTWTQNHVQKLWGPLRNTR----RNGRPNNAVAVVYPPVAVRELEQEVEV
NCU06779.1	GASVDVVMTNSTWTQAHIEKLWGPVRNLTGAVPGIKSKVNPIAVVYPPVAVEELEQEVEV
FG10858.1 	GSSVDIVMTNSTWTQGHIKSLWGPYRIEK-------SKTDPITVVYPPTAVREMENEVEV
AN5725.1  	GGHIDVVMCNSSWTSAHIRSIWGPSRHTP-FLSSKTLKYKDPVVVFPPTAVSDIQSTIPV
          	*. :*:** **:**. *:..:*** *      .      .  .**:**.** :::. : *

MG03883.1 	SVDSESRREKALVYIAQFRPEKNHQLIIQAFAEFVK----SGSDVAKG---------ATL
NCU06779.1	SPESEKRRENVLLYIAQFRPEKNHQLIVQAFAEFLK----SGSEAARD---------AKL
FG10858.1 	SEESEKRREKAIVYLAQFRPEKNHQLILRSFATFVK----TKSEAAKG---------ANL
AN5725.1  	TPETETTRTPTLLYIAQFRPEKNHPLILRSFARFLERHKKTKDSSTKDSSTKKQKPEPQL
          	: ::*. *  .::*:********* **:::** *::  ..: .. ::.:::....... *

MG03883.1 	VLIGSVRDDS-DSKRVYQLRLLVNELHIKDRVEFHLDAPWPRILEWLRKASVGVNGMWNE
NCU06779.1	VLVGSVRDDY-DSKRVYKLRLLVNELHIKDRVEFHLDASWPDILEWLRRASVGVNGMWNE
FG10858.1 	ILIGSVRDDS-DAKRVYQLRLLANELGIKNSVKFHLDAPWSEVLDWLRKASVGVNGMWNE
AN5725.1  	ILIGTVRPSSPDETHIYNLRLLAHELRIRNNTTFLCDASWPTILSHLGTASIGVNAMWNE
          	:*:*:** . .* .::*:****.:** *:: . *  **.*. :*. *  **:***.****

MG03883.1 	HFGIGVVEYQAAGLISVVHDSGGPKLDIVTEIDG-------------EPTG1FHATTSTE
NCU06779.1	HFGIGVVEYQAAGLISVVHDSGGPKLDIVVEVDG-------------EPTG1FHATTSKE
FG10858.1 	HFGIGVVEYQAAGLISVVHDSAGPKFDIVVPIDG-------------QPTG1FHATTEEE
AN5725.1  	HFGICVVEYQAAGLIAVVHDSGGPREDIVIDLPESESGADSLESKGTRATG~FRATTEEE
          	**** **********:*****.**: ***  :  :.:.:.: .:..:..** *:***. *

MG03883.1 	FAEGFRKALSHPN--PLAIRQRARKSAKRFTEEEFAKKWVDQMEKLVALC---------
NCU06779.1	FAEGFEKALSLPN--PYAVRLRARKSAKRFTEEEFARRWIEQLEKCVAIKAVEKPKSRQ
FG10858.1 	FAQGYEKALSLPD--PLAVRLRARESAKRFTEEEFAKKWTVQMARLVTLARREA-----
AN5725.1  	FADAFEKALSLSNDEKVGMRLRARKSALRFTEEEFSRKWVGEIGKLVGMTH--------
          	**:.:.**** .:..  .:* ***:** *******:::*  :: : * :
```
